# Multiple Roles of Exosomal Long Noncoding RNAs in Cancers

**DOI:** 10.1155/2019/1460572

**Published:** 2019-07-07

**Authors:** Wenyuan Zhao, Yuanqi Liu, Chunfang Zhang, Chaojun Duan

**Affiliations:** ^1^Department of Oncology, Xiangya Hospital, Central South University, Changsha, China; ^2^Institute of Medical Sciences, Xiangya Hospital, Central South University, Changsha, China; ^3^Department of Thoracic Surgery, Xiangya Hospital, Central South University, Changsha, China; ^4^National Clinical Research Center for Geriatric Disorders, Xiangya Hospital, Central South University, Changsha, China

## Abstract

Long noncoding RNAs (lncRNAs) are not transcriptional noise, as previously understood, but are currently considered to be multifunctional. Exosomes are derived from the internal multivesicular compartment and are extracellular vesicles (EVs) with diameters of 30–100 nm. Exosomes play significant roles in the intercellular exchange of information and material. Exosomal lncRNAs may be promising biomarkers for cancer diagnosis and potential targets for cancer therapies, since they are increasingly understood to be involved in tumorigenesis, tumor angiogenesis, and chemoresistance. This review mainly focuses on the roles of emerging exosomal lncRNAs in cancer. In addition, the biogenesis of exosomes, the functions of lncRNAs, and the mechanisms of lncRNAs in exosome-mediated cell-cell communication are also summarized.

## 1. Introduction

Noncoding RNAs (ncRNAs) account for the majority of transcribed RNA. Long noncoding RNAs (lncRNAs) are ncRNAs that are larger than 200 nucleotides [1, 2]. Rather than being transcriptional noise, lncRNAs regulate biological activities in a variety of ways, including transcriptional regulation, posttranscriptional regulation, translation regulation, and protein cell localization. LncRNAs are also found to play a necessary role in the progression and prognosis of tumors [3, 4]. LncRNAs have important regulatory functions in fundamental pathological and biological processes, which helps to elucidate the use of lncRNAs and their corresponding proteins or peptides for cancer diagnosis and therapy [5]. EVs play an important role in different disease processes, including renal disease [6], osteoarthritis [7], coronary artery disease [8], dermatology [9], and neurodegenerative diseases [10], leukemia [11] and even have immune-modulatory effects on pregnancy and preeclampsia [12]. In addition, EVs are closely related to endothelial damage in sickle-cell disease [13], sinusoidal obstruction syndrome [14], and essential thrombocythemia [15]. The exosome is a kind of vesicle secreted by living cells that has a diameter of 30-100 nm and a bilayer lipid membrane structure. Exosomes are widely present in biological fluids, such as peripheral blood, ascites, urine, saliva, synovial fluid, and cerebrospinal fluid, as well as bronchoalveolar lavage and breast milk [16]. Exosomes can deliver functional molecules, including lipids, proteins, and nucleic acids, to recipient cells. Exosomes participate in intercellular communication and affect various physiological and pathological functions of cells. For example, pancreatic cancer-derived exosomes are involved in the proliferation, progression, and metastasis of pancreatic cancer [17]. However, the mechanisms by which these exosomal elements affect target recipient cells have not been determined to date. Exosomal lncRNAs have been found to participate in the regulation of tumorigenesis, tumor angiogenesis, and drug resistance, which suggests that there are ample opportunities to explore the potential roles of exosomes as biomarkers in cancer therapies. This review summarizes lncRNA functions and exosome biogenesis in exosome-mediated cell-cell communication and specifically focuses on the emerging roles of exosomal lncRNA in cancer. The EVs studied in some articles reviewed have morphological features of exosomes. However, the term EVs was used in these articles since exosomes are a specific subset of vesicles with a distinctive biogenesis.

## 2. LncRNAs

**Figure 1:**
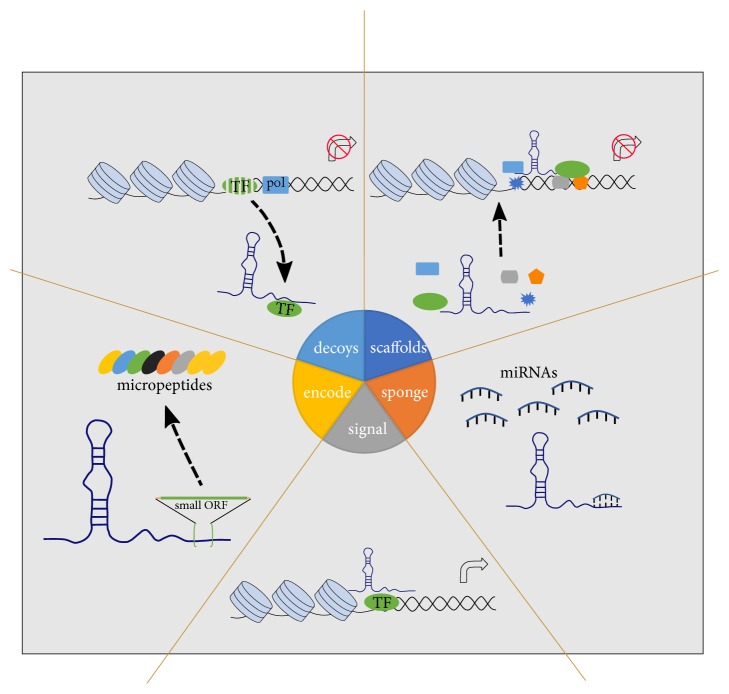
*Functions of lncRNA*. Decoys: lncRNAs act as decoys to attract transcription factors and influence protein expression [23]. Scaffolds: LncRNAs regulate gene expression by recruiting chromatin modifiers to special genomic locations acting as miRNA sponges [9, 10]. Sponge: lncRNAs can interact with miRNA, acting as “sponges” [13]. Signal: lncRNAs have a role in signal regulation [26]. Encode: lncRNAs encode functional micropeptides encoded by short open reading frames [27, 28].

Currently, increasing evidence suggests that lncRNAs have considerable effects on various molecular mechanisms. Prior studies have indicated that mutations of the noncoding genome are widely involved in common human diseases [18]. Regulatory DNA mutations can widely affect transcription by altering enhancer and promoter activity or chromatin states, which leads to the differential expression of lncRNAs in cancer [19]. Although once considered to be transcriptional noise, lncRNAs exhibit various functions, as illustrated in Figure 1, lncRNAs regulate mRNA selective splicing and stability [20]. Additionally, many lncRNAs regulate gene expression by recruiting chromatin modifiers to special genomic locations, similar to scaffolds [21, 22], or by isolating chromatin modifiers from their regulatory locations, similar to decoys [23]. Moreover, lncRNAs control posttranscriptional regulation by functioning as ceRNAs (competing endogenous RNAs) [24] or miRNA sponges [25]. LncRNAs can also directly interact with important signaling proteins (e.g., phosphorylation) and modulate their functions [26]. Some lncRNAs encode functional micropeptides by small open reading frames (smORFs) [27, 28]. More importantly, Pang Y found several peptides which correspond to nine transcripts annotated as ncRNAs [5]. In addition, two smORFs, which were mainly found in ncRNAs and 5' untranslated regions (UTRs), could bind several ribosomes and participate in translation. Dysregulated lncRNAs have been reported to be involved in regulating the proliferation, metastases, and recurrence of multiple cancers, including lung cancer [29], prostate cancer [30], hepatocellular cancer [31], and ovarian cancer [32].

## 3. Exosomes

### 3.1. Exosome Formation

**Figure 2:**
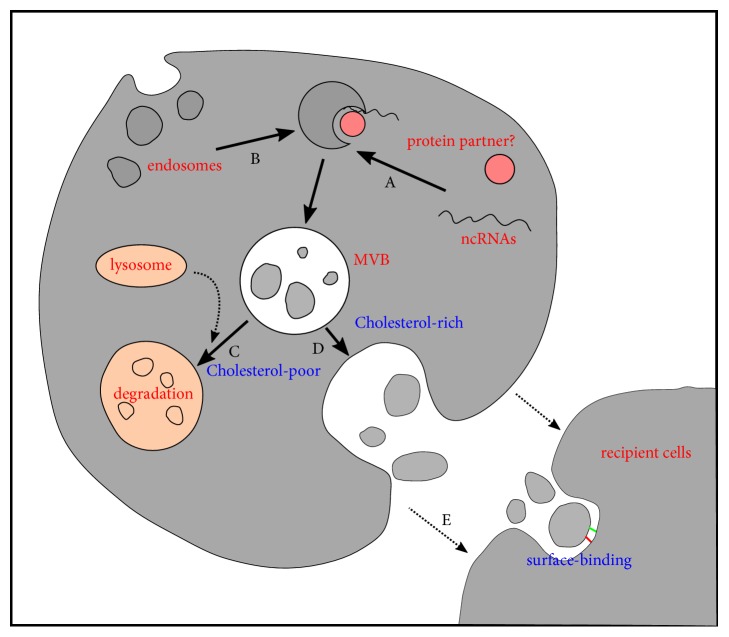
Exosome biogenesis and exosome-mediated delivery of ncRNAs to the recipient cell [37]. A. ncRNAs bind to packing proteins and are selectively secreted. B. Early endosomes are generated from inward budding of the plasma membrane and mature after interacting with Golgi complexes. C. Late endosomes form intraluminal vesicles (ILVs) and incorporate nucleic acids. D. MVB containing ILVs then fuse with the plasma membrane and release exosomes. E. ncRNAs are transferred within exosomes to recipient cells and affect functions.

Exosome biogenesis is observed in various cells, including immune cells, mesenchymal stem cells, neurons, epithelial cells, and endothelial cells (ECs). This process is unlike the formation of microvesicles, which are generated via outward budding at the plasma surface [33]. The underlying mechanism of exosome formation includes several steps. First, an endosome forms through the inward budding of the plasma membrane. Then, further inward budding of the limiting membrane inside the endosome leads to the formation of the multivesicular body (MVB) with a diameter of 30-100 nm, peripheral proteins, cytosolic contents, and the transmembrane, which can be merged into the invaginating membrane through the exocytosis pathway and maintained as extracellular vesicles. MVBs rich in cholesterol fuse with the plasma membrane and then release their contents into the extracellular space. Otherwise, MVBs with deficient cholesterol fuse with lysosomes, causing the degradation of vesicular contents [34]. These released vesicles are known as exosomes. MVB packing was thought to be highly conserved. However, MVB packing is now related to the endosomal sorting complexes required for transport (ESCRT) complex proteins [35]. ESCRT-0, -I, and -II are responsible for recognizing and hiding ubiquitinated membrane proteins in endosomal membranes, and ESCRT-III facilitates cutting and inward budding [36]. However, researchers have observed ESCRT-independent MVB packaging pathways [37] (Figure 2).

### 3.2. Exosomal Molecular Components

Exosomes contain proteins, RNAs, and DNAs [38]. According to the database [ExoCarta (http://www.exocarta.org)], 9769 proteins, 3408 mRNAs, 2838 miRNAs, and 1116 lipids have been identified in exosomes. Extracellular vesicles (EVs) are composed of a lipid bilayer with transmembrane proteins that enclose cytosolic proteins and RNAs [1]. According to the subcellular origin, EVs include microvesicles (100-1000 nm) and exosomes (30-100 nm), which are derived from the internal MVBs [3]. Employing asymmetric flow field-flow fractionation, researchers identified three exosome subgroups: large exosome vesicles (Exo-L, 90-120 nm), small exosome vesicles (Exo-S, 60-80 nm), and “exomeres” (nonmembranous nanoparticles, ~35 nm). Each subpopulation contains a unique component distribution [39]. Metabolic enzymes and hypoxia, microtubule and coagulation proteins, as well as proteins associated with specific pathways, i.e., glycolysis and mTOR signaling, are abundant in exomeres. The proteins contained in Exo-S and Exo-L are involved in endosomal functions, secretion pathways, the mitotic spindle, and IL-2/STAT5 signaling pathways. Additionally, diverse organ distribution patterns have also been observed among those three subpopulations.

### 3.3. Exosomal Release and Transportation

Intracellular calcium, Rab GTPases, and SNARE proteins are crucial elements in exosome release. However, the precise coordination of events involved in exosome release has not been determined [40–42]. Rab27A, Rab27B, and Rab11 were observed to participate in MVE docking at the plasma membrane and to act as mediators in exosome releases [43, 44]. Another six small GTPases are also associated with secretions (Rab2B, Rab5, Rab7, Rab9A, Rab35, and RAL) [16, 45]. SNARE proteins may participate in the fusion of MVEs with the plasma membrane to release ILVs as exosomes [46]. Ca2+ was observed to be involved in the activation of SNARE complexes in many cell types [47]. However, the precise coordination involved in this event has not been determined.

After being released into the extracellular space, extracellular exosomes can be taken up by the recipient cell membrane, thereby delivering exosomal contents into the cytoplasm. In 2007, Valadi et al. first found that exosomes can function as molecular component cargos after they cocultured HMC-1 human mast cells with exosomes isolated from MC/9 murine mast cells [48]. These researchers found that some RNAs exist in vesicles and can be translated by receptor cells. This exosome-mediated intercellular communication requires several steps: first, exosomes binding to the plasma membrane; second, surface receptor and signaling activation; third, vesicle internalization or fusing with the recipient cells [49]. This binding seems target cell-specific and may be determined by proteins enriched between the exosomal surface and the recipient cell plasma membrane [50]. Several mediators of these interactions are known, including extracellular matrix, tetraspanins [51], heparin sulfate proteoglycans [52], and lectins [53].

Exosomes with different compositions may have different functions. An example of this phenomenon is that the *β*-amyloid protein present in exosomes derived from neuroblastoma can be specifically internalized by neurons. However, CD-63-enriched exosomes can bind both neurons and glial cells [54]. Additionally, some special structures at the target cell plasma membrane can influence exosome destiny [55]. Once bound to recipient cells, exosomes can be internalized by endocytosis, phagocytosis, or micropinocytosis [56]. After uptake by recipient cells, exosomes fuse with plasma membrane and release their contents or reach MVBs and undergo digestion by lysosomes [57], whereas some exosomes may escape digestion [58].

### 3.4. Roles of Exosomes in Cancers

Neighboring or distant cells can communicate through the secretion of exosomes. A variety of biological components have been detected in exosomes, such as proteins, mRNAs, and noncoding RNAs [59]. Recent studies have found that tumor-derived EVs participate in promoting antitumor immune responses, helping metastatic dissemination, creating a microenvironment [60], and assisting tumor angiogenesis [61].

## 4. Exosomal lncRNAs

Exosomes contain various ncRNAs, including lncRNAs. Exosomal lncRNAs can be released from cancer cells and internalized by recipient cells, which induces various effects. RNA sequencing shows that exosomal RNAs reflect the intercellular RNA compositions, which suggests that the RNAs are selectively packed into exosomes [62]. Moreover, it has been found that exosomal secretions of RNAs show discrepancies between cancer cells and normal cells [63]. In addition, researchers have observed that lncRNAs with low expression levels in cells are enriched in secreted exosomes [64]. These findings suggest that tumor cells can secrete specific lncRNA-enriched exosomes and may effectively influence recipient cells, which further affects tumorigenesis. In addition to tumorigenesis, exosomal lncRNAs also influence brain disorders [65] and cardiovascular diseases [66]. Accumulating evidence has shown that lncRNAs can be packed into vesicles and detected, which enables circulating lncRNAs to serve as biomarkers [67, 68].

### 4.1. LncRNAs Sorted into Exosomes

The exosomal sorting of RNAs has proven to be highly selective and exhibits cell specificity [69]. Additionally, researchers have noticed that lncRNA molecules contained in exosomes can reflect the cellular response to stimulation, such as DNA damage. These findings suggest a potential regulatory mechanism of sorting ncRNAs into exosomes. However, the mechanism behind packaging specific biological contents into exosomes is not well-understood at present. Researchers found a specific sequence (GGAG) contained in the exosomal miRNAs, which is identified as the EXOmotif and can be specifically recognized by hnRNPA1 (heterogeneous ribonucleoprotein A1) and hnRNPA2B1, thereby regulating the specific loading of such miRNAs into exosomes [70]. Recently, hnRNPA2B1 has also been found to participate in the sorting of lncRNAs into exosomes by recognizing a specific sequence [71]. Another protein, Y-box–binding protein 1 (YBX1), may also help to sort special RNAs into exosomes via binding to specific structural motifs of RNAs, such as UAAUCCCA and CAGUGAGC of lncRNAs and mRNAs [72].

## 5. Functions of Exosomal lncRNAs in Cancers

Exosomal lncRNAs can be used as cancer biomarkers and are strongly involved in tumorigenesis, cancer drug resistance, hypoxia signaling, and EMT. These functions of exosomal lncRNAs are listed in Table 1 according to cancer type and are described in the following subsections in detail.

### 5.1. Cancer Biomarker

The specific lncRNAs contained in cancer cell-derived vesicles may be the measurable and noninvasive clinic biomarkers [73]. Moreover, exosomes prevent proteins and RNAs from being degraded, which renders them intact and functional [74]. In articles published to date, exosomal lncRNAs related to cancer diagnoses and prognoses account for most items.

Serum lncRNAs are commonly used in cancer detection. LncARSR (Ensembl: ENST00000424980) is highly expressed in the plasma of renal cell carcinoma (RCC) patients. In addition, the level of plasma lncRNA-ARSR is decreased after tumor resection and elevated again upon tumor relapse. Correlations between plasma lncRNA-ARSR and progression-free survival (PFS) of RCC patients who underwent sunitinib therapy have also been observed [60]. Exosomal ZFAS1 expression levels are elevated in gastric carcinoma patients and associated with lymphatic metastasis and TNM stage [75]. In addition, with high diagnostic sensitivity and specificity (80.0% and 75.7%), exosomal ZFAS1 is a promising biomarker for gastric cancer diagnosis. Exosomal lncRNAs also exhibit the ability to serve as biomarkers for colorectal adenoma [76, 77], laryngeal squamous cell carcinoma [78], non-small-cell lung cancer [69], and cholangiocarcinoma [71].

In addition to serum, exosomal lncRNAs exacted from other bodily fluids were also found to be plausible biomarkers. Exosomal lncRNA MALAT1, HOTAIR, and MEG3 are differentially expressed in cervical cancer cervicovaginal lavage samples, which suggests that these lncRNAs can be promising biomarkers in detecting cervical cancer [79]. In addition, several lncRNAs (HOTAIR, HOX-AS-2, MALAT1, SOX2, OCT4, HYMA1, LINC00477, LOC100506688, and OTX2-AS1) are enriched in urine exosomes (UEs) from urothelial bladder cancer (UBC) patients [80].

Despite various reports of exosomal lncRNAs functioning as tumor biomarkers, several of these studies did not determine the sensitivity and specificity of the lncRNAs when applied to patients. In addition, many of the studies cannot define the direct relationships of the tested exosomal lncRNAs and cancers. Moreover, methodological differences in EV purification make this approach inadequate in achieving testing reproducibility.

### 5.2. Tumorigenesis

As mentioned earlier, the expression and function of lncRNAs are associated with various types of cancers [81]. Considering that the roles of lncRNAs in cancer are largely unexplored, research on exosomal lncRNAs is still in its infancy. Most studies investigate the roles of different lncRNAs in tumorigenesis, but they fail to demonstrate that the intercellular transfers of lncRNAs via exosomes play roles in tumorigenesis. For example, Iempridee et al. [82] found that lncRNA-H19 enhances the proliferation and spheroid forming ability of cervical cancer cells and is enriched in cell-derived EVs. Similar experiments performed by Kogure et al. show that lncRNA-TUC339 is most highly expressed in hepatocellular carcinoma cells secreting EVs. Up- or downregulation of TUC339 can effectively influence HCC cell proliferation and metastasis [83]. However, these studies did not find direct evidence to demonstrate that exosomes/lncRNAs can directly affect tumorigenesis.

Lei et al. [75] found that lncRNA-ZFAS1 enriched in exosomes can endow recipient cells (low lncRNA-ZFAS1 expression) with increased proliferation and migration ability, which suggests that ZFAS1 can be delivered by exosomes to promote gastric cancer progression.

Dysregulation of angiogenesis occurs in various pathologies and is one of the hallmarks of cancer [84]. Some studies have illustrated that cancer cell-derived exosomes can affect HUVECs in tube formation, in which exosomal lncRNAs may play a pivotal role. CD90+ hepatic cell carcinoma (HCC) has been described with cancer stem-cell-like (CSC) properties [85]. Conigliaro et al. [86] found that exosomes released by CD90+ cancer cells can affect HUVECs by promoting cell-cell adhesion and tube formation. These researchers further found that lncRNA-H19 is enriched in those exosomes. Another study performed by Wu et al. [87] first showed that exosomes isolated from tumor-associated macrophages (TAMs) can incorporate into HUVECs and block the miR146b-5b/TRAF6/NF-*κ*B/MMP2 pathway, which results in efficient reduction of HUVEC migration. In addition, these researchers used SKOV3-derived exosomes and TAM-derived exosomes to costimulate HUVECs and found that inhibition of migration caused by TAM-derived exosomes is overcome. Two exosomal lncRNAs (ENST00000444164, ENST00000437683) were identified as NF-*κ*B pathway-associated genes. A study conducted by Lang et al. [88] found that exosomes enriched in lncRNA-POU3F3 promote angiogenesis in gliomas. Moreover, exosomal lncRNA-POU3F3 has better function in inducing human brain microvascular endothelial cell (HBMEC) migration, proliferation, tube formation, and elevated angio-related gene expression. These results suggest that lncRNAs carried by exosomes can partly influence angiogenesis and further affect tumorigenesis.

### 5.3. Hypoxia Signaling and EMT

Hypoxia in cancer pathology is considered to be a significant element. Tumor cells frequently utilize hypoxia signaling to maintain the proliferative response in normoxia and escape growth arrest in hypoxia [89]. Takahashi et al. first revealed that lncRNA-ROR is a hypoxia-responsive lncRNA and can promote the survival of cancer cells under ischemic conditions. More importantly, these researchers found that lncRNA-ROR can modulate intercellular responses to hypoxia via the transfer of extracellular vesicles. In addition, hypoxia signaling often stimulates a cellular epithelial-mesenchymal transition (EMT) process, which is a critical regulator of metastasis. Several exosomal lncRNAs have been shown to affect EMT signaling in cancer cells. Xue et al. [90] found that UMUC2 has a positive effect on cell proliferation, migration, and invasion when incubated with hypoxic 5637 cell-derived exosomes. Moreover, compared to the normoxic cell-derived exosomes, lncRNA-UCA1 is enriched in hypoxic cell-derived exosomes. These hypoxia-derived lncRNA-UCA1-enriched exosomes can elevate tumorigenesis, both* in vivo* and* in vitro*, and induce cell EMT transformation. Transforming growth factor (TGF)-*β* can promote epithelial-mesenchymal transition (EMT) and further induce invasion and metastasis in pancreatic cancer [91].

### 5.4. Drug Resistance

LncRNAs can be transported by exosomes and endow the recipient cells with acquired drug resistance. Some studies have demonstrated that lncRNAs have potential functions in delivering drug resistance in recipient cells. TGF-1 has been shown to be involved in obtaining chemoresistance in various human cancers [92]. The groups of Takahashi found that lncRNA-ROR and lncRNA-VLDLR can be selectively enriched in EVs by TGF*β*1-stimulated HCC [93]. HCC-derived exosomes can endow HepG2 cells with increased lncRNA-ROR expression and high chemoresistance. Additionally, these researchers found that lncRNA-ROR knockdown can reverse TGF*β*-induced chemoresistance in cancer stem-cell-like CD133+ cells [94]. Another study performed by this team also revealed that lncRNA-VLDLR increases in cells and their EVs under chemotherapeutic stress [95]. These researchers found that lncRNA-VLDLR can be transferred by HCC cell-derived EVs and can promote chemoresistance in recipient cancer cells. Xu et al. [96] found that lncRNA-UCA1 shows high expression in both tamoxifen-resistant LCC2 cells and their derived exosomes. LCC2-derived exosomes facilitate the breast cell line MCF-7 with an increased ability to resist tamoxifen. Moreover, knocking down UCA1 in exo/LCC reverses this phenomenon.

The above studies have proven that exosomal lncRNAs may function in drug resistance; however, they fail to reveal the underlying mechanism of acquired drug resistance related to exosomal lncRNAs. Other articles may better explain the roles of exosomal RNAs in drug resistance. Zhang et al. [97] demonstrated that curcumin-treated cell-derived EVs can reduce the ability of A2780cp cells to induce chemoresistance. LncRNA-MEG3 showed the greatest upregulation in exosomes after curcumin treatment. MEG3 overexpression after curcumin treatment can clearly inhibit miR-214 expression in cells and EVs. These researchers proved that MEG3 can strengthen EV-mediated transfer of miR-214, thereby downregulating drug resistance in recipient cells. These researchers found direct evidence proving that lncRNA-ARSR can be secreted from sunitinib-resistant cells to sensitive cells and induce sunitinib resistance. Intracellular lncRNA-ARSR elevation is directly due to exosome fusion, rather than an increase in intracellular synthesis. LncRNA-ARSR elevation caused by exosomal delivery functions as competing endogenous RNA for miR-449 and miR-34 to facilitate AXL and c-MET expression, which further affects sunitinib resistance.

## 6. Conclusion

In general, exosomes are secreted in almost all types of cells. Exosomes can selectively carry various elements and function as cell-to-cell carriers. LncRNAs secreted by exosomes also play an essential role in cancers. Liquid biopsy through exosomal lncRNAs provides a novel method for diagnosing cancer. Additionally, extracellular lncRNAs packed by exosomes help us evaluate the prognoses and therapeutic effects of the cancers. Moreover, exosomal lncRNAs have been determined to participate in inducing drug resistance in recipient cells, which provides a potential method of cancer therapy. Despite significant progress made in recent years, more work is needed to achieve a better understanding of exosomal lncRNAs in the function and regulation of tumorigenesis.

## 7. Perspective

LncRNAs have shown their utility in the diagnosis and prognosis of some cancers. Unlike commonly used cell-free DNAs (cfDNAs), which originate from dying cells, exosomal nucleic acids (exoNAs), which are derived from living cells, can better reflect the underlying cancer biology [98]. Recently, researchers have presented a novel EGFR T790M assay based on exosomal cfDNAs and RNAs/DNAs from plasma and achieved 92% sensitivity and 89% specificity [99]. However, the use of lncRNAs as biomarkers for cancer diagnosis and prognosis remains limited. First, different methods of isolation, mainly ultracentrifugation-based isolation and exosome precipitation techniques, were used in the aforementioned studies. The methodological differences in exosome isolation and lncRNA extraction make the experimental results difficult to compare. Second, only a small number of lncRNAs have already been investigated, and many of them have been functionally characterized. The construction of an extravascular lncRNA database has greater potential for the study of exosomes.

Moreover, as the natural transporter of functional small RNAs and proteins, exosomes have been suggested to have potential applications in the drug delivery field. It has been demonstrated that specific lncRNAs enriched in exosomes can change the phenotypes of neighboring cells [100]. Moreover, lncRNAs delivered by exosomes can induce drug resistance and angiogenesis in recipient cells. In the field of other exosomal RNAs, researchers have found that MSC-derived exosomes inhibit breast cancer growth by downregulating vascular endothelial growth factor (VEGF) and transferring miR-16 in mice [101]. Additionally, in the field of lncRNAs, intercellular transfer of lncRNA-ARSR through exosomes can significantly dampen the response of RCC xenografts to sunitinib, with increased lncRNA-ARSR expression being observed in tumors. A phase II trial has recently evaluated IFN*γ*-DC-derived exosomes loaded with MHC I/II confined cancer antigens as maintenance immunotherapy after chemotherapy in advanced patients without tumor progression, and exosomes may be used as anticancer vaccines in the future. However, the modulation of lncRNAs* in vivo* is not easy to achieve; therefore, there have been no lncRNA drugs brought into clinical trials to date.

## Figures and Tables

**Table 1:** Overview of exosomal LncRNAs in cancers.

Cancer type	LncRNA	Source	Function	Related genes	Mechanism	Reference
Hepatocellular Carcinoma	Lnc TUC339	Cell	Tumorigenesis	None	Up or down regulation of TUC339 can effectively influence HCC cell proliferation and metastasis	[83]
	Lnc H19 ©	Cell	Tumorigenesis	None	Exosomes released by CD90+ cancer cells can affect HUVECs by promoting tube formation and cell-cell adhesion	[86]
	Lnc-ROR	Cell	Chemoresistance	None	Lnc-ROR can be selectively enriched in extracellular vesicles by TGF*β*1 stimulated HCC/ HepG2 cells	[93]
	Lnc-ROR ©	Cell	Tumor cell ischemia	MiR-145–HIF-1*α*	Lnc-ROR can modulate intercellular responses to hypoxia via the transfer of extracellular-vesicle.	[99]
	Lnc VLDLR	Cell	Chemoresistance	None	Lnc-VLDLR can be transferred by HCC cell derived EVs and promote chemoresistance in recipient cancer cells	[95]

Lung Cancer	MALAT-1 ©	Serum	Biomarker	None	Serum exosomal MALAT-1 was positively associated with tumor stage and lymphatic metastasis	[69]

Gastric Cancer	Lnc 00152 ©	Serum	Biomarker	None	Serum exosomal lnc 00152 was significantly elevated in gastric cancer patients	[64]
	ZFAS1 ©	Serum/Cell	Biomarker/ tumorigenesis	None	ZFAS1 enriched exosomes can endow recipient cell with proliferation and migration	[75]
	HOTTIP ©	Serum	Biomarker	None	Potential biomarker for GC in diagnosis and prognosis	[101]

Colorectal Cancer	CRNDE-h ©	Serum	Biomarker	None	CRNDE-h specificity discriminates CRC patients from NC and benign disease group with high sensitivity	[77]
	Lnc-PVT1	Cell	Potential biomarker	C-Myc	Lnc-PVT1 shows higher expression in more aggressive colorectal cancer cell line	[89]
	KRTAP5-4, MAGEA3 BCAR4	Serum	Potential biomarker	None	Serum exosomal KRTAP5-4, MAGEA3 and BCAR4 provided the greatest predictive ability for colorectal cancer.	[76]

Prostate Cancer	ELAVL1 and RBMX ©	Cell	RNA binding protein binding	None	N/A	[102]

Cervical Cancer	LncRNA MALAT1, HOTAIR, MEG3 ©	Cervicovaginal lavage	Biomarker	None	RT-PCR in identify different expression lncRNA in cervicovaginal lavage	[79]
	H19	Cell/ Serum	Biomarker	Tumorigenesis	H19 promotes cell proliferation and multicellular tumor spheroid formation	[82]

Ovarian cancer	Lnc-MEG3	Cell	Drug resistance	MiR-214	Enriched in curcumin treated cell/ mediated cisplatin resistance	[97]
	ENST00000444164, ENST00000437683 ©	Cell	NF-*κ*B phosphorylation	MiR146b-5b/TRAF6/NF-*κ*B/MMP2	Activating the phosphorylation of NF-*κ*B in HUVECs and further affecting tumorogenesis	[87]

Colon Cancer	LncRNA AC007193.8, RUSC1-AS1, TM4SF1-AS1, DLGAP1-AS1, DLGAP1-AS1, SETD5-AS1, DNAJC27-AS1 TTC28-AS1 ©	Cell	None	None	Different lncRNAs enricher in extracellular vesicle subtypes	[103]

Glioma	Lnc-POU3F3 ©	Cell	Endothelial cell angiogenesis	BFGF, VEGFA, bFGFR, and Angio	Lnc-POU3F3 enriched exosomes may induce HBMEC migration, proliferation, and tube formation	[88]

Bladder Cancer	HOTAIR, HOX-AS-2, MALAT1, SOX2, OCT4, Lnc HYMA1, LINC00477, LOC100506688 and OTX2-AS1©	Urine	Biomarker	None	Potentially serving as biomarkers for UBC diagnosis	[80]
	Lnc-UCA1 ©	Cell	Hypoxic resistance/biomarker	HIF-1*α*, p27, miR-143	Hypoxic derived lnc-UCA1 enriched exosome can elevate tumorigenesis and induce cell EMT transformation	[90]
	Lnc-UCA1 ©	Cell	Drug resistance	HIF-1*α*, p27, miR-143	Lnc-UCA1 increases the tamoxifen resistance	[96]

laryngeal squamous cell carcinoma	HOTAIR ©	Serum	Biomarker	None	Diagnose combing serum exosomal miR-21 and HOTAIR can have achieve good sensitivity and specificity	[78]

Renal Cancer	LncARSR ©	Serum/Cell	Biomarker/ Drug resistance	HnRNPA2B1, AKT/FOXO axis, miR-34a, miR-449	LncARSR can be specifically packed into exosomes via hnRNPA2B1; LncARSR enriched exosomes can induce sunitinib sensitivity with resistance.	[60]

Cholangiocarcinoma	ENST00000588480.1, ENST00000517758.1 ©	Bile	Biomarker	None	N/A	[71]

© refers to the articles which confirmed the usage of the term exosome. Other articles used the term EVs instead although the EVs studied in these articles have morphological features of exosomes.
